# Directional local field potentials: A tool to optimize deep brain stimulation

**DOI:** 10.1002/mds.27215

**Published:** 2017-11-18

**Authors:** Gerd Tinkhauser, Alek Pogosyan, Ines Debove, Andreas Nowacki, Syed Ahmar Shah, Kathleen Seidel, Huiling Tan, John‐Stuart Brittain, Katrin Petermann, Lazzaro di Biase, Markus Oertel, Claudio Pollo, Peter Brown, Michael Schuepbach

**Affiliations:** ^1^ MRC Brain Network Dynamics Unit at the University of Oxford Oxford United Kingdom; ^2^ Nuffield Department of Clinical Neurosciences University of Oxford United Kingdom; ^3^ Department of Neurology Bern University Hospital and University of Bern Bern Switzerland; ^4^ Department of Neurosurgery Bern University Hospital and University of Bern Bern Switzerland; ^5^ Neurology Unit Campus Bio‐Medico University of Rome Rome Italy; ^6^ Department of Neurosurgery University Hospital Zurich, University of Zurich Zurich Switzerland

**Keywords:** Parkinson's disease, deep brain stimulation, directional leads, local field potentials, DBS programming

## Abstract

**Background:** Although recently introduced directional DBS leads provide control of the stimulation field, programing is time‐consuming.

**Objectives:** Here, we validate local field potentials recorded from directional contacts as a predictor of the most efficient contacts for stimulation in patients with PD.

**Methods:** Intraoperative local field potentials were recorded from directional contacts in the STN of 12 patients and beta activity compared with the results of the clinical contact review performed after 4 to 7 months.

**Results:** Normalized beta activity was positively correlated with the contact's clinical efficacy. The two contacts with the highest beta activity included the most efficient stimulation contact in up to 92% and that with the widest therapeutic window in 74% of cases.

**Conclusion:** Local field potentials predict the most efficient stimulation contacts and may provide a useful tool to expedite the selection of the optimal contact for directional DBS. © 2017 The Authors. Movement Disorders published by Wiley Periodicals, Inc. on behalf of International Parkinson and Movement Disorder Society.

A major advance in DBS technology was the introduction of directional DBS leads with segmented contacts and multiple source current steering.^1^, ^2^ The middle two levels of conventional ring‐contact DBS electrodes are replaced with three segmented (noncircular) contacts (Fig. 1A), which allow steering of the stimulation field. Two intraoperative studies,^1^, ^3^ a postoperative clinical trial and a case report,^4^, ^5^ have reported an increased therapeutic window and efficacy of directional compared to spherical stimulation.

**Figure 1:**
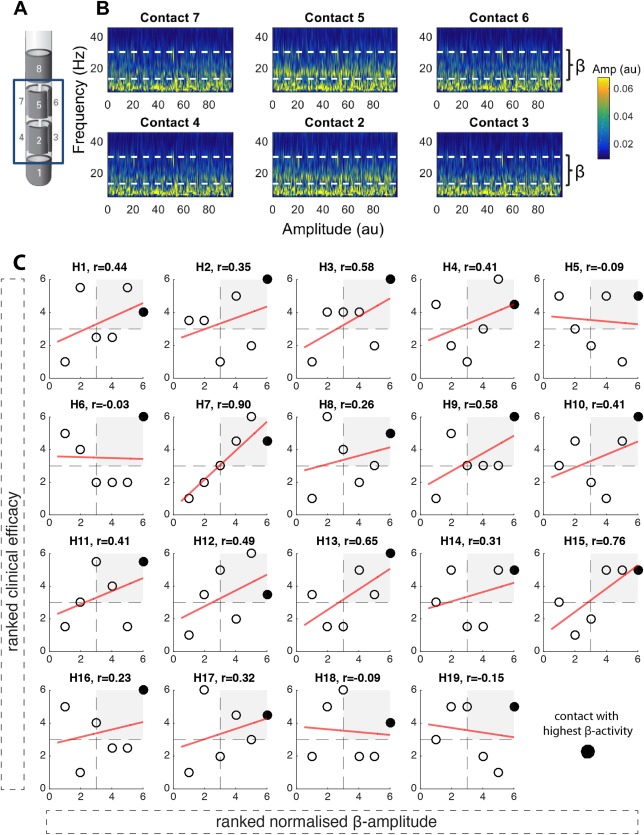
Directional LFPs and relationship between ranked beta activity and clinical efficacy. (A) illustrates the directional DBS lead (Boston Scientific, Marlborough, MA). Contacts are distributed along four levels. On levels two and three, there are three segmented contacts (level two: contacts 2/3/4; level three: contacts 5/6/7). (B) shows an example time frequency spectrum from an intraoperative LFP recording (duration, 100 seconds) from the six directional contacts (2/3/4; 5/6/7) with the patient awake and at rest. The dashed white line marks the beta frequency band (13‐35 Hz). It shows that LFP beta activity is not equally distributed across directional contacts. Contact 5 shows the highest beta activity, followed by contact 2, with both contacts 5 and 2 oriented in the same direction. Data from the right hemisphere in subject 3 (for raw data, amplitude‐frequency spectrum, and imaging from the same subject and hemisphere, see Supplementary Fig. 1). (C) illustrates the relationships between normalized beta activity and clinical efficacy across the six directional contacts in each hemisphere (H = hemisphere; n = 19). The normalized beta amplitude is shown on the x‐axis, the clinical efficacy on the y‐axis, and Spearman correlation coefficients are shown on the top of each panel. The best electrophysiological contact (contact with highest normalized beta activity) is highlighted in black. The red linear regression fit is shown only for illustration purposes. In 15 hemispheres, a positive relationship between clinical efficacy and normalized beta activity was found (*t*_18_ = 4.65; *P* < 0.001, one‐sample *t* test). In 12 of 19 hemispheres, the contact with the highest beta activity matched the clinically most effective stimulation contact. Furthermore, in all hemispheres, the contact with the highest beta activity was localized in the upper‐right quadrant, where the clinically more efficient contacts are localized. Clinical efficacy and normalized beta activity are illustrated as ranked values; Supplementary Figure 3 shows the same figure with nonranked values. [Color figure can be viewed at wileyonlinelibrary.com]

These advantages are offset by the complexity of programming directional DBS. The monopolar contact review is the crucial initial step and gold standard for the management of DBS patients^6^, ^7^ and requires a highly trained person.^8^ The use of directional DBS leads implies testing of a total of 16 stimulation contacts (8/hemisphere), with 12 (6/hemisphere) of them being segmented (Fig. 1A) and requires much more time. Thus, tools that can expedite programming and optimize the use of directional electrodes are strongly needed.^9^

Local field potential (LFP) activity in the beta band (13‐35 Hz) has previously been shown to be related to motor symptoms in Parkinson's disease (PD) and to predominantly arise in the motor portion of the STN.^10^, ^11^, ^12^ Importantly, it has also been demonstrated that the ring contact closest to the beta source is clinically more efficient compared to other contacts.^11^, ^13^, ^14^, ^15^ Here, we test the hypothesis that delivery of stimulation in the direction of the highest beta‐band activity in the STN provides the best stimulation effect and that the LFP may therefore serve as a tool to assist DBS programming.

## Patients and Methods

Twelve PD patients undergoing STN‐DBS surgery were implanted with directional leads (Boston Scientific, Marlborough, MA; Supplementary Table 1). LFPs were recorded during surgery from the directional contacts after each lead was placed in its final position (Fig. 1B). Normalized beta activity was derived from each directional contact by normalization of the individual beta peak activity by the whole beta band (13‐35 Hz). In cases where no beta peak was present, the low beta band (13‐20 Hz) was normalized. Monopolar contact review took place 4 to 7 months postsurgery. Clinical efficacy (% rigidity improvement/stimulation current) and therapeutic window (TW) were calculated for each directional contact and compared with the corresponding normalized beta activity. Detailed methods are included in the Supplementary Material.

## Results

### Relationship Between Beta Activity and Response to stimulation

In 15 of 19 hemispheres tested, we found a positive relationship (*t*_18_ = 4.65; *P* < 0.001, one‐sample *t* test) between normalized beta activity and clinical efficacy (ranked values: Fig. 1C; absolute nonranked values: Supplementary Fig. 3). Thus, the higher the relative beta activity recorded from a specific directional contact, the better its clinical efficacy. In all cases, the contact with highest beta was consistently one of those with higher clinical efficacy, and in 12 of 19 cases (63%), it corresponded to the contact with the highest clinical efficacy. In 7 of 19 cases (36%; hemispheres 13‐19), we did not find a clear beta peak, but despite this, the relationship was similar to those with a clear peak in the beta band. There was also no difference in the predictive value of the level and orientation of the beta peak between those hemispheres with a beta peak up to 20 Hz (=2, 3, 5, 6, 7, 10, and 11) and those hemispheres with a beta peak above 20 Hz (hemispheres 1, 4, 8, 9, and 12). The ring level containing the contact with the highest beta activity was localized in the dorsal STN (ventral STN = 0; middle STN = 4; dorsal STN = 15).

### Predictive Value of Beta Activity for the Most Efficient Stimulation Contact

**Figure 2:**
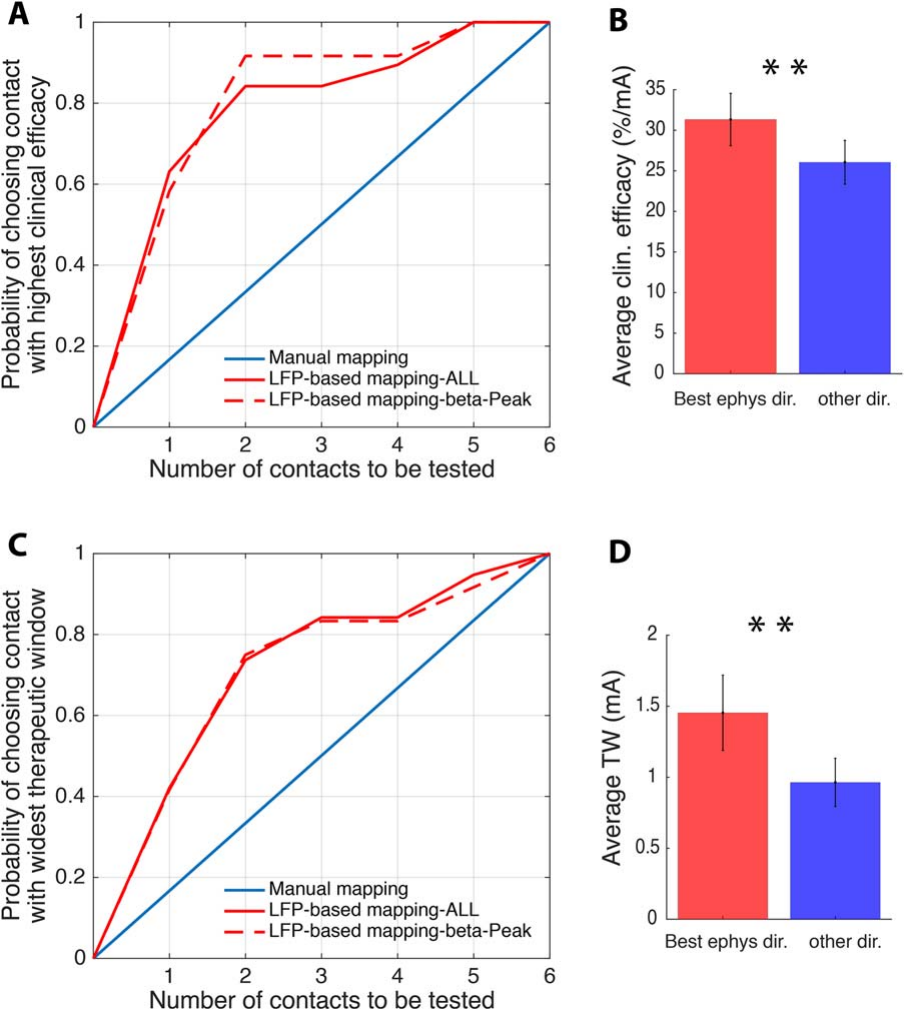
LFP‐based DBS programming. (A) **s**hows the probability of identifying the stimulation contact with the highest clinical efficacy, comparing the conventional (random) test strategy in blue with the LFP‐based test strategy in red (full red line: all hemispheres n = 19, dashed red line: only hemispheres with clear beta peak n = 12). While for conventional mapping the probability of identifying the most efficient stimulation contact increases by 0.17 with each contact tested, the LFP‐based strategy identifies the most efficient contact with a probability of 0.63 if only the contact with the highest beta activity is considered, and with a probability of 0.84 if the two contacts with the highest beta activity are considered. By considering hemispheres with a clear beta peak only, the probability increases up to 0.92 when the two best electrophysiological contacts are considered. (B) The mean clinical efficacy of the two directional stimulation contacts with the highest beta activity (“Best ephys”) is significantly higher compared to the clinical efficacy of the remaining directional contacts (“other dir.”). (C) (similar to A) shows the probability of identifying the contact with the highest therapeutic window by again comparing the conventional (random) test strategy in blue with the LFP‐based test strategy in red (full red line: all hemispheres n = 19; dashed red line: only hemispheres with clear beta peak n = 12). While for conventional mapping the probability of identifying the stimulation contact with the widest therapeutic window increases by 0.17 with each contact tested, the LFP‐based strategy identifies the contact with the widest therapeutic window with a probability of 0.42 if only the contact with the highest beta activity is considered, and with a probability of 0.74 if the two contacts with the highest beta activity are considered. No relevant difference in the predictive value is found when exclusively hemispheres with a clear beta peak are considered (dashed red line). (D) The mean TW of the two directional stimulation contacts with the highest beta activity (“Best ephys”) is significantly higher compared to the therapeutic window of the remaining directional contacts. Values are mean ± SEM; **P < 0.01. [Color figure can be viewed at wileyonlinelibrary.com]

In Figure 2A, we tested the predictive value of contacts ranked by relative beta power for clinical efficacy. This shows that the stimulation contact with the highest beta activity was able to predict the stimulation contact with the highest clinical efficacy in 63% of cases. More strikingly, when including the contact with the second‐highest beta activity, the prediction rose to 84%, and up to 92% if only hemispheres with a clear beta peak (n = 12) were considered. In contrast, conventional clinical testing had only a 17% likelihood of identifying the most efficient contact if only one contact was assessed, and a 34% likelihood if two contacts were assessed. Figure 2B shows that the mean clinical efficacy of the two contacts with the highest beta activity was significantly higher (31.3 ± 3.2%/mA [milliamperes]) compared to the mean clinical efficacy (26.1 ± 2.7%/mA) of the remaining contacts of the same electrode (*t*_18_ = 3.75; *P* = 0.0015, paired *t* test).

### Relationship Between Clinical Efficacy and Therapeutic Window

Another important clinical parameter is the therapeutic window, which also includes the side‐effect threshold. Figure 2C shows that the LFP‐based strategy identified the contact with the widest therapeutic window in 42% of cases if only the contact with the highest beta activity was considered, and in 74% if the two highest beta contacts were considered. No relevant difference in the predictive value was found when hemispheres with a clear beta peak were exclusively considered. Additionally, Figure 2D shows that the mean therapeutic window of the two contacts with the highest beta activity was significantly higher (1.45 ± 0.27 mA) compared to the mean TW (0.96 ± 0.17 mA) of the remaining contacts of the same electrode (*t*_18_ = 3.11; *P* = 0.006, paired *t* test).

## Discussion

In this study, we demonstrate, in a sizeable patient cohort, that the two segmented contacts of the directional DBS electrode with maximal STN beta activity are highly likely to include the contact that turns out to have the best efficacy with a wide therapeutic window. Clinical testing was performed at least 4 months after lead implantation, when the majority of any stun effect has lapsed^16^ and the clinical relevance of contact screening therefore heightened. Thus, the LFP can serve as a predictive and supportive tool for multicontact lead programming. This is in line with previous studies showing similar results for the ring contact electrode,^11^, ^13^, ^14^, ^15^ as well as with an intraoperative trial^17^ and a single, early postoperative case report with directional stimulation.^18^

Why should beta power in the LFP predict the clinical efficacy of stimulation fields of different orientation? It has been shown that the dorsal part of STN is the most effective site for STN stimulation in PD,^19^, ^20^ and that this is also the focus of beta activity.^11^, ^12^, ^13^ Yet, the LFP cannot afford direct information about the contact specific therapeutic window, because side effect threshold depends on the vicinity of the stimulation field to neighboring structures. On the other hand, as current directed to the dorsal STN is less likely to spread to these neighboring areas, the prediction of the contact with the lowest threshold for clinical effect may also explain the predictive value for the contact with the widest therapeutic window.

### LFP‐Based Programming

If we assume that it takes around 20 minutes to assess stimulation at each contact, then monopolar contact review of segmented leads will take around 4 hours (12 segmented contacts). This would be fatiguing for both clinician and patient, leading to variability in assessments. If only those two segmented contacts that have the highest beta activity are screened on each side, then there is approximately a 90% probability of selecting the contacts that have the lowest effect threshold. This would only take 80 minutes, reducing assessment time by approximately two thirds. Moreover, as discussed above, the two segmented contacts with the highest beta activity are also more likely to have a wider therapeutic window. Hence, this method potentially offers a physiologically based, time‐saving approach to the programming of directional electrodes.

### Limitations

In this investigation, we only studied those sides with at least two points of upper‐limb rigidity and more than a minimum range of responses to stimulation across contacts. We also limited the electrophysiological‐clinical comparison to the clinical data acquired during the monopolar review session, where rigidity was the only systematically assessed item. However, rigidity is also the most sensitive clinical sign to DBS.^6^ These inclusion criteria were chosen to optimize the clinical comparison across contacts, and to avoid ceiling and floor effects. The value of beta activity and of other LFP features in predicting the best contact for tremor suppression needs further evaluation.

In addition, we assumed that the monopolar review in and of itself is predictive of chronic stimulation settings.^6^, ^19^ Moreover, manual clinical contact testing, although the current “gold standard” for determining the best stimulation contacts, is a subjective method with some degree of inter‐rater variability. Any noise in the gold‐standard estimation will only have served to degrade the apparent predictive value of the LFP.

Intraoperative time constraints meant that LFPs could only be recorded for around 2 minutes (with interindividual variability), and longer recordings might have been more representative. Importantly, we also assumed that lead position and orientation did not change after LFP recording. Furthermore, our data may have been contaminated by stun effects, which can be detected as the STN is traversed and lead to diminished beta power.^10^

### Future Directions and Conclusion

Tools that can assist DBS programming by the clinician or even run fully automatically are desirable in this era of directional, multicontact leads. This could streamline the postoperative management of patients, and free up clinical resources to contend with the increasing numbers of such patients dictated by growing experience with this therapy and by the move to offer DBS earlier during the disease course.^21^ Nevertheless, the clinical advantage, or lack thereof, of chronic directional DBS still needs to be definitively demonstrated.

The method presented is of potential predictive value with respect to subsequent programming, regardless of whether microelectrode recordings are used or not in targeting the STN. In time, optimal contact prediction might be based on a variety of features. The electrophysiological approach taken here might be supplemented by radiological‐anatomical strategies that could provide more‐accurate information about surrounding structures. However, presently, these are challenging to implement, given that target structures are small and image resolution is limited, so that the error rate in lead localization is not negligible.^22^

In conclusion, the present study suggests that the amplitude of subthalamic beta LFP activity is predictive of the most efficient stimulation contact and can form the basis for a rapid programming tool useful for multicontact directional DBS leads.

## Author Roles

G.T., P.B., S.M., and C.P. conceptualized the study; G.T. and P.B. drafted the manuscript; G.T. and A.P. conducted signal processing and main analyses. S.A.S., H.T., L.d.B., and J.‐S.B. provided important intellectual input. I.D., K.P., and S.M. performed the clinical testing. I.D., K.P., K.S., S.M., and G.T. performed intraoperative recordings. A.N. and C.P. performed radiological validation. C.P., M.O., and A.N. performed the surgery.

## Financial Disclosures

Nothing to report.

## Supporting information

Additional Supporting Information may be found in the online version of this article at the publisher's web‐site.

Supplementary Information

## References

[1] Pollo C , Kaelin‐Lang A , Oertel MF , et al. Directional deep brain stimulation: an intraoperative double‐blind pilot study. Brain 2014;137:2015‐2026. 24844728 10.1093/brain/awu102

[2] Timmermann L , Jain R , Chen L , et al. Multiple‐source current steering in subthalamic nucleus deep brain stimulation for Parkinson's disease (the VANTAGE study): a non‐randomised, prospective, multicentre, open‐label study. Lancet Neurol 2015;14:693‐701. 26027940 10.1016/S1474-4422(15)00087-3

[3] Contarino MF , Bour LJ , Verhagen R , et al. Directional steering: a novel approach to deep brain stimulation. Neurology 2014;83:1163‐1169. 25150285 10.1212/WNL.0000000000000823

[4] Steigerwald F , Müller L , Johannes S , Matthies C , Volkmann J . Directional deep brain stimulation of the subthalamic nucleus: a pilot study using a novel neurostimulation device. Mov Disord 2016;31: 1240‐1243. 27241197 10.1002/mds.26669 PMC5089579

[5] Reker P , Dembek TA , Becker J , et al. Directional deep brain stimulation: a case of avoiding dysarthria with bipolar directional current steering. Parkinsonism Relat Disord 2016;31:156‐158. 27591075 10.1016/j.parkreldis.2016.08.007

[6] Volkmann J , Moro E , Pahwa R . Basic algorithms for the programming of deep brain stimulation in Parkinson's disease. Mov Disord 2006;21:284‐289. 10.1002/mds.20961 16810675

[7] Picillo M , Lozano AM , Kou N , et al. Programming deep brain stimulation for Parkinson's disease: The Toronto Western Hospital Algorithms. Brain Stimul 2016;9:425‐437. 26968806 10.1016/j.brs.2016.02.004

[8] Moro E , Poon YY , Lozano AM , et al. Subthalamic nucleus stimulation: improvements in outcome with reprogramming. Arch Neurol 2006;63:1266‐1272. 16831958 10.1001/archneur.63.9.1266

[9] Kühn AA , Volkmann J . Innovations in deep brain stimulation methodology. Mov Disord 2017;32:11‐19. 27400763 10.1002/mds.26703

[10] Chen CC , Pogosyan A , Zrinzo LU , et al. Intra‐operative recordings of local field potentials can help localize the subthalamic nucleus in Parkinson's disease surgery. Exp Neurol 2006;198:214‐221. 16403500 10.1016/j.expneurol.2005.11.019

[11] Zaidel A , Spivak A , Grieb B , Bergman H , Israel Z . Subthalamic span of beta oscillations predicts deep brain stimulation efficacy for patients with Parkinson's disease. Brain 2010;133(Pt 7):2007‐2021. 20534648 10.1093/brain/awq144

[12] Horn A , Neumann WJ , Degen K , Schneider GH , Kühn AA . Toward an electrophysiological ‘sweet spot’ for deep brain stimulation in the subthalamic nucleus. Hum Brain Mapp 2017 Apr 8. doi: 10.1002/hbm.23594. [Epub ahead of print] 10.1002/hbm.23594 PMC6867148 28390148

[13] Yoshida F , Martinez‐Torres I , Pogosyan A , et al. Value of subthalamic nucleus local field potentials recordings in predicting stimulation parameters for deep brain stimulation in Parkinson's disease. J Neurol Neurosurg Psychiatry 2010;81:885‐889. 20466699 10.1136/jnnp.2009.190918

[14] Ince NF , Gupte A , Wichmann T , et al. Selection of optimal programming contacts based on local field potential recordings from subthalamic nucleus in patients with Parkinson's disease. Neurosurgery. 2010;67:390‐397. 20644424 10.1227/01.NEU.0000372091.64824.63 PMC4319368

[15] Connolly AT , Kaemmerer WF , Dani S , et al. Guiding deep brain stimulation contact selection using local field potentials sensed by a chronically implanted device in Parkinson's disease patients. In: International IEEE/EMBS Conference on Neural Engineering, NER (Vol. 2015‐July, pp. 840‐843). [7146754] IEEE Computer Society. doi: 10.1109/NER.2015.7146754.

[16] Mestre TA , Lang AE , Okun MS . Factors influencing the outcome of deep brain stimulation: placebo, nocebo, lessebo, and lesion effects. Mov Disord 2016;31:290‐298. 26952118 10.1002/mds.26500

[17] Bour LJ , Lourens MA , Verhagen R , de Bie RM , van den Munckhof P , Schuurman PR , Contarino MF . Directional recording of subthalamic spectral power densities in Parkinson's disease and the effect of steering deep brain stimulation. Brain Stimul 2015;8:1‐12. 25753176 10.1016/j.brs.2015.02.002

[18] Fernández‐García C , Foffani G , Dileone M , Catalán‐Alonso MJ , González‐Hidalgo M , Barcía JA , Alonso‐Frech F . Directional local field potential recordings for symptom‐specific optimization of deep brain stimulation. Mov Disord 2017;32:626‐628. 28339118 10.1002/mds.26949

[19] Herzog J , Fietzek U , Hamel W , et al. Most effective stimulation site in subthalamic deep brain stimulation for Parkinson's disease. Mov Disord 2004;19:1050‐1054. 15372594 10.1002/mds.20056

[20] Pollo C , Vingerhoets F , Pralong E , et al. Localization of electrodes in the subthalamic nucleus on magnetic resonance imaging. J Neurosurg 2007;106:36‐44. 10.3171/jns.2007.106.1.36 17240554

[21] Schuepbach WM , Rau J , Knudsen K , et al. Neurostimulation for Parkinson's disease with early motor complications. N Engl J Med 2013;368:610‐622. 23406026 10.1056/NEJMoa1205158

[22] Nestor KA , Jones JD , Butson CR , et al. Coordinate‐based lead location does not predict Parkinson's disease deep brain stimulation outcome. PLoS One 2014;9:e93524. 24691109 10.1371/journal.pone.0093524 PMC3972103

