# Supplementary material for: Directional local field potentials: A tool to optimize deep brain stimulation
Source: Mov Disord. 2017 Nov 18;33(1):159–64. doi: 10.1002/mds.27215 (PMC5768242; doi:10.1002/mds.27215)
Supplement: Supplementary file 1 — Supplementary Information [file MDS-33-159-s001.docx]

# Methods

## Patients and Surgery

Twelve patients (19 hemispheres) diagnosed with PD (8 male, 4 female; age 49-76 years; disease duration 5.8-13.2 years; pre-operative MDS-UPDRS III off levodopa: 40±2.6, on levodopa: 14.7±1.1) were selected for STN DBS surgery and were implanted with the new directional electrodes and the Vercise PC (Boston Scientific). See supplemental table 1 for further details. The DBS target was localised using the T2-sequence of the pre-operative 3T MRI and preoperative stereotactic CT-Scan (with Leksell G frame). Intraoperative targeting was optimised by microelectrode recording and selective test stimulation. The lead orientation was assessed using anterior-posterior and lateral X-rays and a postoperative brain CT scan with the frame in place was obtained immediately after surgery and fused with the pre-operative imaging. The electrode position was determined by referencing to the mid commissural point. The STN was further subdivided along its ventral-dorsal axis into a ventral, middle and dorsal segment. This segmentation began with reconstruction (normalisation) of the images along the AC PC line. Then the longest extension of the STN in the normalised axial orientation was divided into three equally long segments. Thereafter each of the electrode ring levels in the STN was attributed to one of these segments. Image processing and validation was performed using Brainlab software (Brainlab AG, Germany). All patients were recruited and operated at the University Hospital Bern. The local ethics committee approved the study (2017-00551).

## Local field potential recording and signal processing

LFPs were recorded during DBS surgery from the directional contacts after placement of the lead in its final position using the *Inomed, ISIS IOM system*. Contacts were arranged in a monopolar configuration with the cannula as common reference. Signals were simultaneously recorded from all contacts in a given hemisphere for up to 3 minutes with the patients awake and at rest. After artefact removal signal duration was on average 101.6s (range 23.2 to 155.9s). The raw signal was sampled at 2000 Hz, but down-sampled to 200 Hz and band pass filtered between 1 and 80 Hz prior to analysis. Amplitude frequency spectra with 1 Hz resolution were calculated using Wavelet transformation (ft_specest_wavelet script in Fieldtrip - Morlet Wavelet, width=6, gwidth=3; Donders Institute for Brain, Cognition and Behaviour, 2010). Beta peak area was defined as the area in the bin containing the peak beta frequency together with that in the neighbouring two bins on either side of this. This was possible for recordings where a visually clear identifiable beta peak was present. Otherwise the area was set to be that of the low beta band (13-20Hz). This frequency range was selected because current literature suggests that the lower beta frequencies (<20Hz) correlate more strongly with PD motor symptoms than higher beta frequencies [15]. The total power of the beta peak area was calculated for each directional contact and normalised with the total power of the beta band (13-35Hz) for that contact. Methodological steps are illustrated in figure 1, and example amplitude frequency spectra are shown in supplementary figure 1.

## Clinical contact testing and parameters

Clinical assessment of stimulation through each of the contacts took place not earlier than 4 months (17 to 31 weeks) after DBS surgery to avoid major DBS stun effects [16] and was performed by the DBS-team, blinded to LFP data. Clinical contact testing of each contact followed standardized monopolar contact review procedures [6] after dopaminergic medication was withdrawn at least 12 hours prior to testing (levodopa was stopped at least 12 hours before, dopamine agonists up to 48 hours before). Clinical assessment focused on upper limb rigidity testing only. First the effect threshold (ET) was determined as the stimulation current necessary to completely relieve rigidity or to obtain the best achievable improvement in this clinical sign. The side effect threshold (ST) was defined as the stimulation current where limiting side effects occurred. Both ET and ST were fine-tuned using smaller current amplitude (mA) steps. Stimulation frequency and pulse width were set to 130Hz and 60µs by default. Therapeutic window (TW) was defined as the difference between ST and ET. DBS efficacy was defined according to the following formula:

$$\frac{100\times(rigidity at baseline - rigidity at ET)}{rigidity at baseline \times Current at ET}$$

Importantly, we applied two clinical inclusion criteria. First, at least 2 points in MDS-UPDRS assessed upper limb rigidity were required at baseline (during stimulation OFF). Second, we required at least 4 different levels of ET across the six directional contacts. Both these steps were necessary to increase the dynamic range of the clinical response and to avoid floor effects. In particular, lack of clinical variability (i.e. too many contacts with identical response to DBS) would have blunted our ability to assess the predictive value of the LFP. These criteria led to the exclusion of 4 hemispheres (3 hemispheres had less than 3 levels of ET, 1 hemisphere had only 1 point of rigidity) and one additional hemisphere could not be included because no systematic directional testing was done (see supplementary table 1).

## Data analysis

Statistical analyses were performed using Matlab (version R 2015b; MathWorks, Natick, MA) and IBM SPSS Statistics (Version 23). Normality of data distribution was validated by visual inspection of the corresponding QQ plots. The relationship between normalized beta activity and clinical efficacy of the contacts for each hemisphere was assessed using Spearman’s correlation, for both ranked and non-ranked data. Whether Fisher transformed r-values differed from zero was tested using a one sampled t-test for the whole group and separately also for hemispheres with lower frequency beta peak (within 13-20 Hz) and higher frequency beta peak (>20Hz). The therapeutic window and clinical efficacy of the two contacts with the highest beta activity was compared with the therapeutic window of the remaining contacts of the same DBS electrode using parametric testing. The cumulative probability of contacts including the most efficient stimulation contact and contact with the widest therapeutic window was assessed starting with the contact with the highest beta activity, and then by consecutively adding the directional contact with the next highest beta and so on. All data are presented as means ± standard error of the mean (SEM).

# Legends, Tables

| **Subject** | **Num.Hem.** | **Site** | **Sex** | **Age at DBS (yr)** | **Disease dur. (yr)** | **Predom. Symptom** | **UPDRS III OFF LD** | **UPDRS III**  **ON LD** | **Beta peak**  **fr.** |
| --- | --- | --- | --- | --- | --- | --- | --- | --- | --- |
| 1 | 1 | L | M | 49 | 6,9 | right akinetic-rigid | 21 | 6 | 22 |
|  | 2 | R |  |  |  |  |  |  | 20 |
| 2 | 3 | L | M | 68 | 10,3 | right akinetic-rigid | 36 | 13 | 17 |
|  | 4 | R |  |  |  |  |  |  | 24 |
| 3 | 5 | L | M | 49 | 6,5 | right akinetic-rigid | 41 | 13 | 16 |
|  | 6 | R |  |  |  |  |  |  | 16 |
| 4 | 7 | L | M | 64 | 16,7 | left akinetic-rigid | 44 | 20 | 17 |
|  | 8 | R |  |  |  |  |  |  | 21 |
| 5* | 9 | L | M | 63 | 5,8 | right akinetic-rigid | 17 | 8 | 22 |
| 6* | 10 | L | F | 68 | 5,8 | right akinetic-rigid, tremor | 44 | 12 | 18 |
| 7* | 11 | R | M | 66 | 5,9 | left akinetic-rigid,tremor | 41 | 16 | 14 |
| 8* | 12 | L | F | 74 | 11,0 | right tremor, akinetic-rigid | 51 | 23 | 25 |
| 9 | 13 | L | M | 76 | 13,2 | right akinetic-rigid | 44 | 19 | 13-20 |
|  | 14 | R |  |  |  |  |  |  | 13-20 |
| 10 | 15 | L | M | 74 | 12,2 | right akinetic-rigid | 55 | 14 | 13-20 |
|  | 16 | R |  |  |  |  |  |  | 13-20 |
| 11 | 17 | L | F | 73 | 7,7 | left akinetic-rigid | 36 | 13 | 13-20 |
|  | 18 | R |  |  |  |  |  |  | 13-20 |
| 12^#^ | 19 | L | F | 50 | 8,7 | right akinetic-rigid | 50 | 19 | 13-20 |
| Mean ± SEM |  | L (11) R(8) | M(8)  F(4) | 64.5±2.3 | 9.2±0.8 |  | 40±2.6 | 14.7±1.1 | 18.3±0.7 |

**Table 1: Clinical details.** Num = number; Hem = hemisphere; R = right; L = left; yr = years; dur = duration; Predom = predominant; UPDRS = Unified Parkinson’s disease rating scale Part III; LD = levodopa; fr = Frequency; SEM = standard error of the mean; *subjects where 1 hemisphere was excluded, because clinical inclusion criteria were not met. # clinical data from right hemisphere are not available.

**
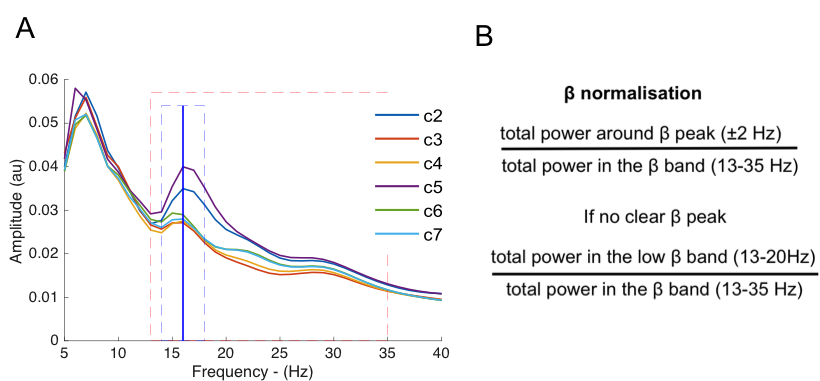
**

**Supplementary figure 1: Exemplary spectra and normalisation method.** **A** shows the time averaged spectra from the six directional contacts (2/3/4; 5/6/7). Contact 5 shows the highest beta activity, followed by contact 2. Both these contacts have the same orientation. Example corresponds to the right hemisphere of subject 3 (see also figure 1B). **B** shows the way we normalised beta activity for each individual directional contact, i.e. total power in beta peak area (area encompassed by the beta frequency peak and 2Hz above and below this) divided by the total power in the beta band (13-35Hz). In cases where no clear beta peak was present (n=7), the beta peak area was defined from 13-20Hz and similarly normalised with the total power in the beta band.


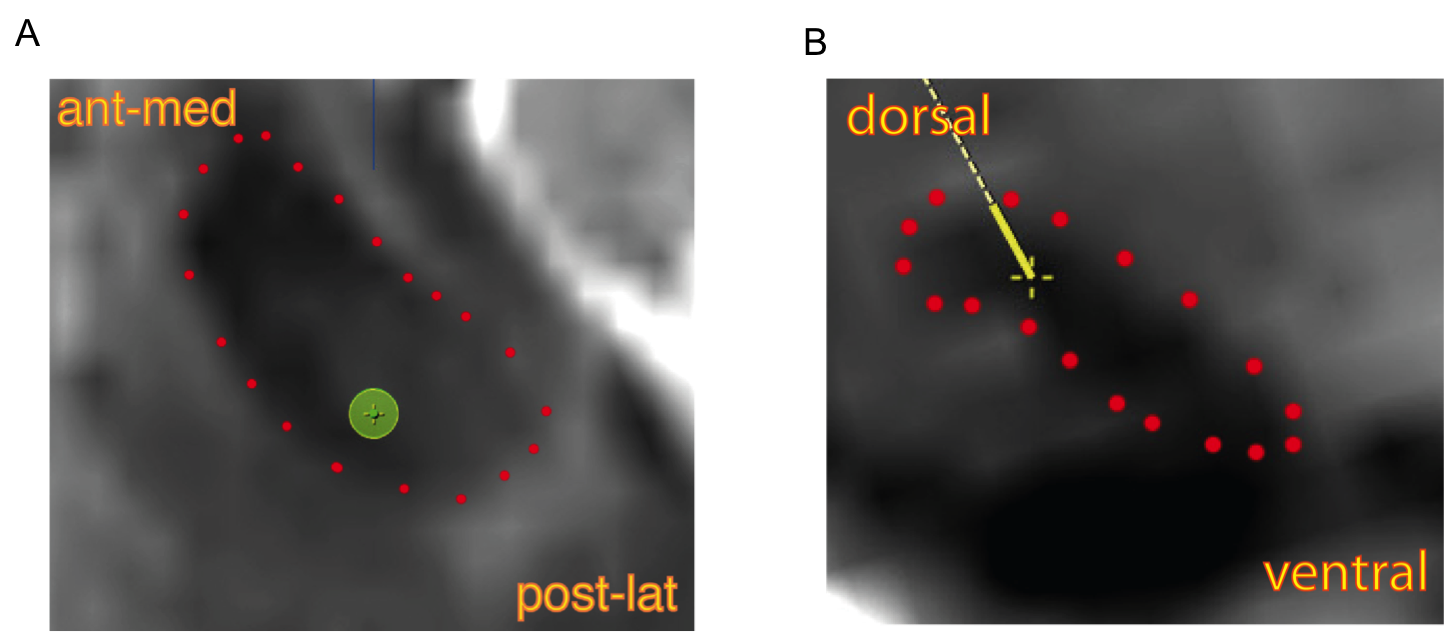


**Supplementary figure 2:** Lead localisation. This illustrates both the axial (A) and sagittal (B) view of the right STN. Both slices correspond to the ring level 3 of the lead, which contains the contact with the highest beta activity, localised in the dorsal motor STN.

**
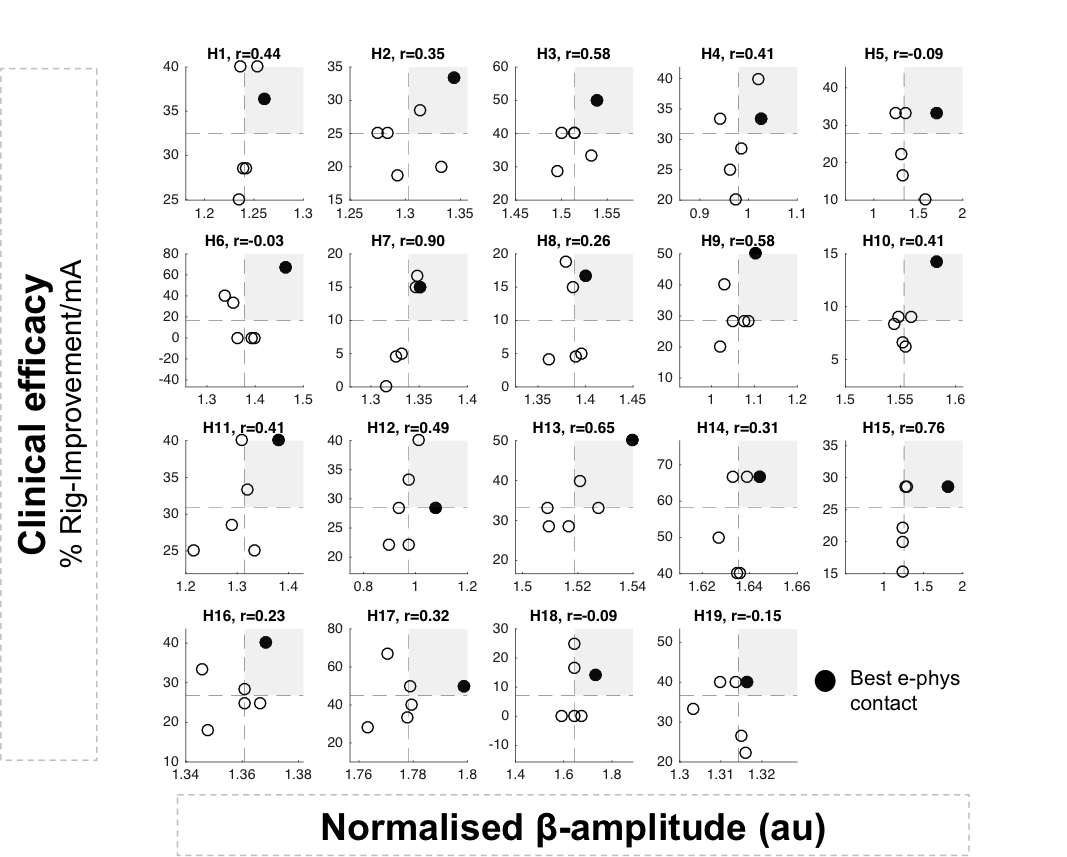
**

**Supplementary figure 3: Relationship between beta activity and clinical efficacy.** Illustrates the relationships between normalised beta activity and clinical efficacy across the 6 directional contacts in each hemisphere (H=hemisphere, n=19). The normalized beta amplitude is shown on the x-axis, the clinical efficacy on the y axis and Spearman correlation coefficients shown at the top of each panel. The best electrophysiological contact (contact with highest normalised beta activity) is highlighted in black. In 15 hemispheres a positive relationship between clinical efficacy and normalised beta activity was found (one sampled t-test, p<0.001). In 12 out of 19 hemispheres the contact with the highest beta activity matched the clinically most effective stimulation contact. Furthermore, in all hemispheres the contact with the highest beta activity was localised in the upper right quadrant, where the clinically more efficient contacts are found.
